# Marked Response of Rat Ileal and Colonic Microbiota After the Establishment of Alzheimer’s Disease Model With Bilateral Intraventricular Injection of Aβ (1-42)

**DOI:** 10.3389/fmicb.2022.819523

**Published:** 2022-02-11

**Authors:** Qing Xu, Lingmiao Wen, Guihua Wei, Xiaoqin Zhao, Yanjun Liu, Wei Xiong, Tinglan Zhang, Yuqing Fan, Chunlan Chen, Chunxiao Xiang, Chang Chen, Yunhui Chen, Qiaozhi Yin, Tian-e Zhang, Zhiyong Yan

**Affiliations:** ^1^School of Life Science and Engineering, Southwest Jiaotong University, Chengdu, China; ^2^Institute of Chinese Materia Medica, China Academy of Chinese Medical Sciences, Beijing, China; ^3^School of Basic Medicine, Chengdu University of Traditional Chinese Medicine, Chengdu, China

**Keywords:** Alzheimer’s disease, β-amyloid (1-42), surgery, ileal microbiota, colonic microbiota, 16S rRNA

## Abstract

Alzheimer’s disease (AD) is a common neurodegenerative disease. More evidence has shown that gut microbiota is closely associated with AD. Also, studies have shown that the distribution of gut microbiota vary in different sections of the intestine. In this study, a rat model of AD was established using a bilateral intraventricular injection of β-amyloid (1-42) [Aβ (1-42)], and the behavior of rats, hippocampal Aβ (1-42) deposition, and the ileal and colonic microbiota in each group were analyzed. We observed that the model rats had obvious memory and cognitive impairment, increased Aβ (1-42) deposition, indicating that the AD model was successfully established. Through 16S rRNA-sequencing analysis, we found that α diversity, β diversity, and dominant microbiota in the ileum and colon of normal rats were significantly different, showing spatial heterogeneity. Additionally, the surgery and injection of Aβ (1-42) caused various degrees of disturbances in the ileal and colonic microbiota of rats. These findings provide new insights for the study of the gut microbiota of AD rats and help advance the development of therapeutic strategies for intervening AD through the gut microbiota.

## Introduction

Alzheimer’s disease (AD) is an age-related neurodegenerative disease. Its main characteristics are β-amyloid deposition and neurofibrillary tangles, and its clinical manifestations begin with slight memory decline and can eventually develop into severe self-care and cognitive impairment (Mathys et al., 2019; Kim et al., 2020). According to the Alzheimer’s Disease International (2016), the number of people with dementia in the world is about 50 million (Alzheimer’s Disease International, 2016). The etiology of AD is still unclear. Some studies have suggested that accumulation of Aβ in the brain is the primary influence driving AD pathogenesis (Gitter et al., 2000; Hardy and Selkoe, 2002), and others show that tau protein hyperphosphorylation leads to neurofibrillary tangles, resulting in neurotoxicity (Iqbal et al., 2016; Wesseling et al., 2020). Also, some studies consider AD as a chronic inflammatory disease of the central nervous system (Sierra-Filardi et al., 2011; Krabbe et al., 2013). In addition, the concept of Brain-Gut-Microbiota Axis has been proposed in recent years and suggests that gut microbiota can influence neurodegenerative disorders (Cryan et al., 2019; Kowalski and Mulak, 2019; Sun et al., 2020). In this study, we established an AD model by bilateral intraventricular injection of Aβ (1-42) based on the β-amyloid cascade hypothesis. This method has been widely used (Yang et al., 2018; Xu et al., 2019), and many studies have shown that Aβ (1-42) injection can cause memory impairment and changes Brain Derived Neurotrophic Factor (BDNF) or 5-hydroxytryptamine(2A)[5-HT(2A)] receptor levels in the serum and brain (O’Hare et al., 1999; Christensen et al., 2008).

Gut microbiota is a complex microbial community that is symbiotic in the intestines of humans or animals. Different microbiota in the intestine maintains the micro ecological balance of the intestine and participates in the body’s digestion and metabolism, biosynthesis of vitamins, immune regulation, energy conversion, and other functions (Chow et al., 2010). There is increasing evidence that gut microbiota affects gut-brain interaction at different points in time (from early life to neurodegeneration) and at different levels (from the intestinal cavity to the central nervous system) (Dinan and Cryan, 2017; Kowalski and Mulak, 2019). Changes in the gut microbiota can act on various neurodegenerative diseases, including Parkinson’s disease, AD, multiple sclerosis, and amyotrophic lateral sclerosis, etc., (Sarkar and Banerjee, 2019). Recently, the new AD drug “GV-971” developed by China (Shanghai Green Valley Pharmaceutical Co., Ltd., Shanghai, China) reshapes the balance of the gut microbiota, inhibits the abnormal increase in specific metabolites of the gut microbiota, reduces the peripheral and central inflammation, and reduces β-amyloid protein deposition and Tau protein hyper phosphorylation, thereby improving patients’ cognitive dysfunction. This is also the first new drug for AD targeting the gut-brain axis (Wang et al., 2019).

The mammalian intestine is divided into different intestinal segments, including the jejunum, ileum, cecum, and colon (Li et al., 2020). As a result of various factors, including the pH value, oxygen content, intestinal peristalsis intensity, etc., there are certain differences in the composition, number, and diversity of colony between different individuals or different intestinal segments of the same individual (Eckburg et al., 2005; Donaldson et al., 2016; Tropini et al., 2017; Martinez-Guryn et al., 2019). In the same body, the number of microbiota gradually increases from top to bottom with the direction of the intestine and is more distributed in the ileum and colon, more especially, the colon is the main site of habitation for bacterial residents with an estimated concentration of 10^12^/ml (Sender et al., 2016). Current studies on gut microbiota mostly use colonic or fecal microbiota as samples (Bhattarai et al., 2021; Jing et al., 2021). Most of the colon or fecal contents contain large intestine microorganisms, but they lack small intestine microorganisms. Therefore, this research focuses on the ileum and colon to explore the composition and changes in the microbiota.

This study established an AD animal model by bilateral intraventricular injection of Aβ (1-42), and the composition of the microbiota in different intestinal segments of normal rats was compared and analyzed. Furthermore, the effects of model-making surgery and injection of Aβ (1-42) on rat ileal and colonic microbiota were analyzed. Present, there are many studies on AD and gut microbiota, but studies on the microbial variation in different sections of the intestine are still rare. This study explores the specific changes in the ileal and colonic microbiota during AD lesions, to promote follow-up research of intervention or treatment of AD using the gut microbiota.

## Materials and Methods

### Animals

A total of 36 specific pathogen free (SPF) adult Sprague–Dawley rats aged 6∼7 weeks, half male and half female, were purchased from Chengdu Dashuo Experimental Animal Co., Ltd., Chengdu, China, and the production license is SCXK (Sichuan) 2015–030. The animals were raised in the Animal Laboratory of the School of Life Sciences and Engineering, Southwest Jiaotong University, and the temperature was maintained at 25°C ± 2°C, alternating light and dark for 12 h, and had access to food and water *ad libitum*. The animal study was reviewed and approved by the Animal Ethics Committee of Southwest Jiaotong University (No. SWJTU-2010-001) and performed in compliance with the Guidelines for Animal Experimentation of the university. After being adaptively reared for 7 days, they were randomly divided into three groups: the normal group, the sham-operated group, and the model group, each with 12 animals, half male, and half female.

### Establishment of Alzheimer’s Disease Animal Model

The AD model was established by referring to the methods of Granic et al. (2010), Xu et al. (2019). Diluted Aβ (1-42) (J0426A, meilunbio) to 2 μg/μL with sterile saline incubated it in a constant temperature incubator at 37°C for 7 days to make it into an aggregated state and then stored it at 4°C. After placed in an induction chamber and anesthetized with 3% isoflurane, the rats in the model group used surgical scissors to clean their head hair and fixed it on the stereotaxic instrument. The skin of the operation area was first disinfected and then the fontanelle was found. With Bregma as the origin, the two-puncture point was located 3.2 mm after Bregma and 2.0 mm to the left and right of Lambda, and the needle of microinjector was inserted vertically 2.9 mm (AP = −3.2 mm, ML = 2.0 mm, DV = 2.9 mm). In the model group, 2-μL Aβ (1-42) was slowly injected into the bilateral hippocampal CA1 area, and the wound was sutured and disinfected after the needle was withdrawn. The rats in the sham-operated group were injected with 2-μL sterile saline, and the normal group was not treated in any way.

### Morris Water Maze Test

One month after the Aβ (1-42) injection, Morris water maze (MWM) test was conducted (Kelley et al., 2011). The experimental system consists of a pool, a movable platform, an image acquisition and analysis system. The pool was divided into four quadrants, and a platform 2-cm below the water surface was set in the third quadrant. The test was divided into two parts: place navigation test and spatial probe test. Briefly, rats were subjected to acquisition training for 4 consecutive days, then place navigation test on the 5th day. The time(s) spent by each rat to locate the platform (the platform incubation period) was recorded. The rats were placed into the water facing the wall of the pool. If they found the platform within 120 s, they were allowed to stay on the platform for 20 s; if they were not found, they were guided to find the platform and stay for 20 s. On the 6th day of the experiment, the platform was removed for the spatial probe test, and the rats were placed in the swimming pool from the same position. The target quadrant dwell time(s) and crossings(n), and the number of platform crossings(n) within the specified time (120 s) were recorded.

### Sampling for Aβ Detection of Hippocampus

After the Morris water maze test, the rats were sacrificed and the hippocampal region of the brain was extracted. The appropriate amount of hippocampal tissue was mixed with nine times the amount of saline and ground into a homogenate. The supernatant was centrifuged at 3,000 rpm for 10 min after which testing was conducted according to the Aβ (1-42) assay kit (6C739NJDT9, Elabscience Biotechnology Co., Ltd., Wuhan, China).

### Sampling for Microbiota Detection

While taking the hippocampal tissue, the contents of the ileum and colon were collected and stored in freezer at −80°C. Four samples were taken from each group, and 24 samples were analyzed using 16S rRNA gene amplicon sequencing. According to the manufacturer’s instructions, Zymo Research BIOMICS DNA Microprep Kit (Cat# D4301) was used for sample DNA extraction and purification. The 16S rDNA V4 region of the samples was then amplified using Applied Biosystems^®^ PCR System 9700, which primer sequences were as follows: Primer 5′-3′: 515F (5′-GTGYCAGCMGCCGCGGTAA-3′) and 806R (5′- GGACTACHVGGGTWTCTAAT-3′) with an amplification length of 292 bp (Chengdu Ronin Biotechnology Co., Ltd., Chengdu, China). And the products were detected, purified, and quantified. Libraries were built using NEW ENGLAND BioLabs NEBNext Ultra II DNA Library Prep Kit for Illumina (NEB# E7645L), and high-throughput sequencing was conducted using the Hiseq 2500 platform in PE250 mode.

### Analysis of Bacterial Microbiota Detection Results

The original sequence of each sample was quality-controlled and filtered using the QIIME (v1.9.0) software package (Caporaso et al., 2010). Based on the Usearch (10.0.240) software, the UPARSE algorithm (Edgar, 2013) was used to conduct operational taxonomic unit(OUT) clustering at a consistency level of 97%, and the sequence with the highest frequency in each OTU was selected as the representative sequence of OTU. The UCLUST classification (Edgar, 2010) is used for annotation analysis, and FastTree (Price et al., 2010) is used to construct phylogenetic tree. The community composition analysis, α diversity, and β diversity analysis were conducted using the R language, which included the Vegan package for Chao1 and Shannon indices analysis, the GuniFrac package to calculate Unifrac distances, the ape package for PCoA analysis, and the ggplot2 package for community composition analysis. Different species analysis was conducted using LEfSe analysis (Segata et al., 2011).

### Data Analysis

The experimental data were analyzed using IBM SPSS Statistics 20 and Graphpad Prism 8.0, and the comparison of sample parameters between groups were conducted using the one-way analysis of variance (ANOVA) followed by least significant difference (LSD) tests and Tukey test for multiple comparisons. The data are all expressed as mean ± Standard Error of Mean ($\bar{\text{x}}$ + SEM), and *P* < 0.05 indicates that the difference is statistically significant.

## Results

### Effect of Surgery and Aβ (1-42) Injection on the Rat Behavior

After modeling, a water maze experiment was conducted to evaluate the spatial memory of rats in the normal, sham-operated, and model groups. According to the results (Table 1), there were no significant differences between the normal and sham-operated groups in platform incubation period, target quadrant dwell time and crossings, number of platform crossings. While the platform incubation period of the model group rats was significantly longer (*F* = 3.986, *P* < 0.05). The target quadrant dwell time (*F* = 7.974, *P* < 0.05, *P* < 0.01) and crossings (*F* = 7.459, *P* < 0.01) and the number of platform crossings (*F* = 8.095, *P* < 0.05, *P* < 0.01) were significantly shortened. It was observed that the injection of Aβ (1-42) caused damage to the memory and cognitive function of rats, but it was not affected by the surgery.

**TABLE 1:** Effect of surgery and Aβ (1-42) injection on rat behavior.

Group	Platform incubation period(s)	Target quadrant dwell time(s)	Target quadrant crossings(n)	Number of platform crossings(n)
Normal group	11.52 ± 2.53	43.603 ± 2.209	45.830 ± 8.185	8.330 ± 1.639
Sham-operated group	12.95 ± 4.08	39.777 ± 2.096	39.080 ± 7.638	5.580 ± 0.557
Model group	29.20 ± 7.04*^#^	31.833 ± 2.071**^#^	12.250 ± 1.256**^##^	2.500 ± 0.399**^#^

Mean ± SEM, n = 12. *P < 0.05, **P < 0.01 compared with the normal group.

*^#^P < 0.05, ^##^P < 0.01 compared with the sham-operated group.*

### Effect of Surgery and Aβ (1-42) Injection on the Content of Aβ (1-42) in Hippocampus of Rats

**FIGURE 1:**
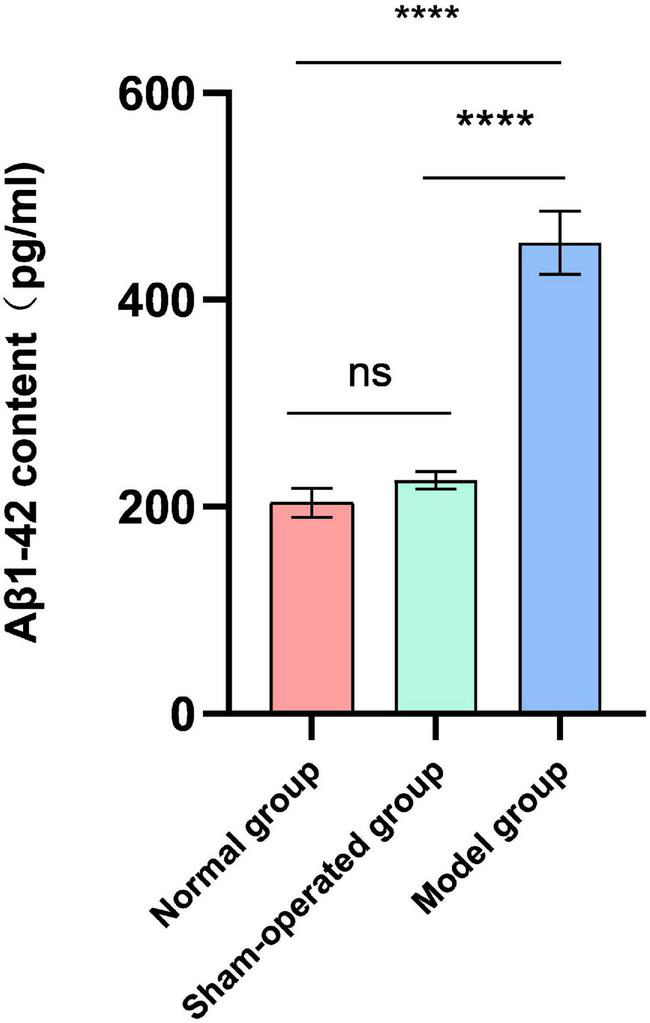
Effect of surgery and Aβ (1-42) injection on the content of Aβ (1-42). *n* = 6. ****P < 0.0001.

After the water maze experiment, the effects of surgery and Aβ (1-42) injection on the content of Aβ (1-42) in the hippocampus of rats were explored. There was no significant difference in the levels of Aβ (1-42) (pg/ml) in the hippocampus of rats in the normal group and the sham-operated group (Figure 1). In contrast, the deposition of Aβ (1-42) in the model group significantly increased (*F* = 48.51, *P* < 0.0001, Figure 1).

### Effect of Surgery and Aβ (1-42) Injection on the Diversity of the Ileal and Colonic Microbiota of Rats

**FIGURE 2:**
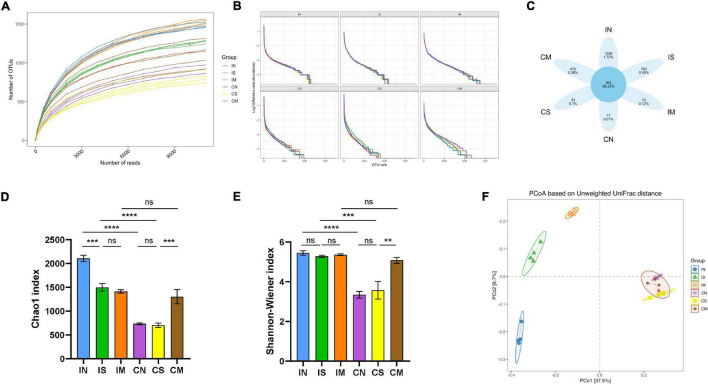
Effect of surgery and Aβ (1-42) injection on the diversity of the ileal and colonic microbiota of rats. **(A)** Rarefaction Curve shows the variation trend of the species richness of each sample with the sequencing depth. **(B)** Rank-Abundance shows the relationship between the abundance of individual species and the type of individual species in each sample. **(C)** Venn diagram shows the OTU unique to each sample and shared by different samples. Chao1 index **(D)** and Shannon–Wiener index **(E)** of α-diversity of 16S rRNA sequencing of each sample in different groups. **(F)** Principal co-ordinates analysis (PCoA) for β-diversity shows the clustering of gut microbial communities in different groups. IN: the ileal microbiota of the normal group; IS: the ileal microbiota of the sham-operated group; IM: the ileal microbiota of the model group; CN: the colonic microbiota of the normal group; CS: the colonic microbiota of the sham-operated group; CM: the colonic microbiota of the model group. **P < 0.01; ***P < 0.001; and ****P < 0.0001.

We read a total of 5,48,256 effective tags from 24 samples, with an average of 22,844 sequence reads per sample for subsequent analysis. As the sequencing depth increases, the species richness of each group of samples increased, and the Rarefaction Curve (Figure 2A) and Rank-Abundance (Figure 2B) are gradually flattening out. The sequencing depth basically covered all species in the sample, and the species distribution in each sample is also relatively uniform. The Venn diagram displayed 1,336 unique OTUs in the IN, 763 unique OTUs in the IS, 70 unique OTUs in the IM, 17 unique OTUs in the CN, 33 unique OTUs in the CS, and 472 unique OTUs in the CM. In total, 202 OTUs are shared in all groups (Figure 2C).

The Chao1 index is commonly used to estimate the total number of species, and the Shannon index is commonly used to assess the number of species and homogeneity of microorganisms. In the normal group and the sham-operated group, the Chao1 index (*F* = 44.34, *P* < 0.0001, Figure 2D) and Shannon index (*F* = 20.67, *P* < 0.0001, *P* < 0.001, Figure 2E) of the ileal microbiota were significantly higher than those of the colonic microbiota; while in the model group, Chao1 and Shannon index in the ileal and colonic microbiota are not statistically different. In the ileum samples (Figures 2D,E), compared with the normal group, the Chao1 index of the sham-operated group was significantly lower (*F* = 44.34, *P* < 0.001). Still, there was no significant difference between the model group and the sham-operated group, and the Shannon index was not significantly different in the three groups. In the colon samples (Figures 2D,E), the two indices of the normal group and the sham-operated group were not significantly different, while the Chao1 (*F* = 44.34, *P* < 0.001) and Shannon (*F* = 20.67, *P* < 0.01) indices of the model group were significantly higher than those of the sham-operated group. The Unweighted Unifrac distance matrix was utilized in analyzing the colony structure between each group of samples, the more similar the community composition of the samples, the closer their distance is in the figure. The results showed (Figure 2F) that the ileal and colonic microbiota in the normal group, sham-operated group, or model group were significantly separated. In the ileum samples, the structure of the microbiota in the normal group, the sham-operated group, and the model group were clearly separated. In the colon samples, the community distribution of the normal group, the sham-operated group, and the model group were relatively clustered. This indicated that the ileal and colonic microbiota of normal rats were significantly different, and the species number and diversity of the ileal microbiota were significantly higher than that of the colonic microbiota. The surgery has significantly reduced the number of species in the ileal microbiota and changed its structure, but had no significant effect on the colon; the injection of Aβ (1-42) changed the colony structure of the ileum and increased the species number and diversity of the colonic microbiota.

### Effect of Surgery and Aβ (1-42) Injection on the Composition of the Bacterial Community in the Ileum and Colon of Rats

**FIGURE 3:**
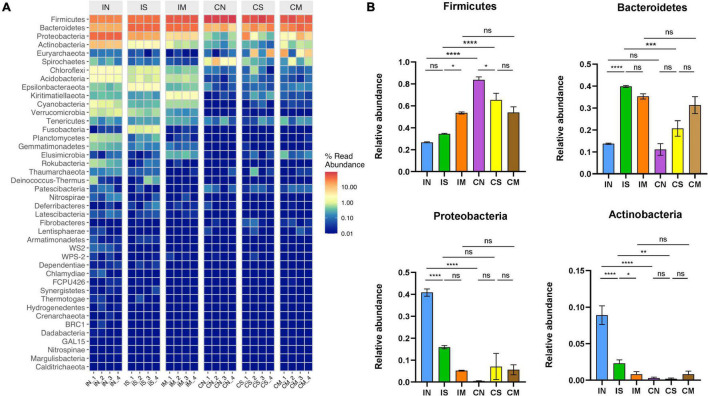
Effect of surgery and Aβ (1-42) injection on the composition of the bacterial community at the phylum level. **(A)** The distribution of the top 50 species with the highest abundance at the phylum level **(B)** the distribution of the top four dominant bacteria phyla with the highest abundance at the phylum level in each group of samples. IN: the ileal microbiota of the normal group; IS: the ileal microbiota of the sham-operated group; IM: the ileal microbiota of the model group; CN: the colonic microbiota of the normal group; CS: the colonic microbiota of the sham-operated group; CM: the colonic microbiota of the model group. Data are reported as the mean ± SEM. *P < 0.05; **P < 0.01; ***P < 0.001; and ****P < 0.0001.

To further explore the changes in the ileal and colonic microbiota during the modeling process, the relative composition of the communities of different intestinal segments at the phylum and genus level were analyzed. At the phylum level (Figure 3), the most important phyla in rat intestines are Firmicutes (from 26.56 to 83.72%), Bacteroidetes (from 11.08 to 39.84%), Proteobacteria (from 0.45 to 40.82%), and Actinobacteria (from 0.19 to 8.91%). In the normal group, the abundance of Proteobacteria (*F* = 29.41, *P* < 0.0001) and Actinobacteria (*F* = 122.3, *P* < 0.0001) in the ileal microbiota was significantly higher than that of the colonic microbiota, and the abundance of the Firmicutes of the colonic microbiota was significantly higher than that of the ileal microbiota (*F* = 35.32, *P* < 0.0001). In the sham-operated group, the abundance of Bacteroidetes (*F* = 23.71, *P* < 0.001) and Actinobacteria (*F* = 122.3, *P* < 0.01) in the ileal microbiota was higher than that of the colonic microbiota, and the Firmicutes in the colonic microbiota was significantly higher than that of the ileal microbiota (*F* = 35.32, *P* < 0.0001). There was no significant difference in the abundance of each dominant phyla in the ileum and colon. Additionally, in the ileum samples, Bacteroidetes in the sham-operated group were significantly increased compared to the normal group (*F* = 23.71, *P* < 0.0001), Proteobacteria (*F* = 29.41, *P* < 0.0001) and Actinobacteria (*F* = 122.3, *P* < 0.0001) were significantly decreased, and the abundance of Firmicutes in the model group was significantly increased compared to the sham-operated group (*F* = 35.32, *P* < 0.05), Actinobacteria abundance decreased significantly (*F* = 122.3, *P* < 0.05). In the colon sample, Firmicutes in the sham-operated group was significantly lower than that in the normal group (*F* = 35.32, *P* < 0.05), but the dominant phyla of the model group had no significant difference compared with the sham-operated group.

**FIGURE 4:**
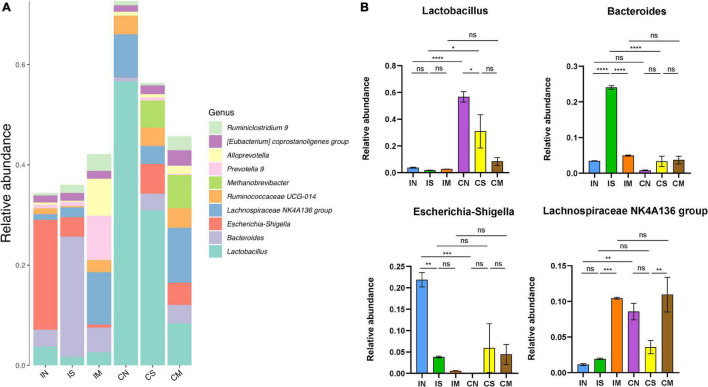
Effect of surgery and Aβ (1-42) injection on the composition of the bacterial community at the genus level. **(A)** The distribution of the top 10 species with the highest abundance at the genus level. **(B)** The abundance distribution of the top four bacterial genera with the highest abundance at the genus level in each group of samples. IN: the ileal microbiota of the normal group; IS: the ileal microbiota of the sham-operated group; IM: the ileal microbiota of the model group; CN: the colonic microbiota of the normal group; CS: the colonic microbiota of the sham-operated group; CM: the colonic microbiota of the model group. Data are reported as the mean ± SEM. *P < 0.05; **P < 0.01; ***P < 0.001; and ****P < 0.0001.

At the genus level (Figure 4), the most important bacterial genera in rat intestines are *Lactobacillus* (from 1.68 to 56.62%), *Bacteroides* (from 0.77 to 24.03%), *Escherichia-Shigella* (from 0.06 to 21.91%), *Lachnospiraceae NK4A136 group* (From 1.13 to 10.94%). In the normal group, the abundance of the colonic microbiota of *Lactobacillus* (*F* = 16.61, *P* < 0.0001) and *Lachnospiraceae NK4A136 group* (*F* = 14.27, *P* < 0.01) were significantly higher than that of the ileal microbiota, while the abundance of the ileal microbiota of *Escherichia-Shigella* was significantly higher than that of the colonic microbiota (*F* = 9.586, *P* < 0.001). In the sham-operated group, the abundance of *Lactobacillus* in the colon was significantly higher than that of the ileal microbiota (*F* = 16.61, *P* < 0.05), and the *Bacteroides* of the ileal microbiota were significantly higher than that of the colonic microbiota (*F* = 124.1, *P* < 0.0001). In the model group, there was no significant difference in the abundance of each dominant genus in the ileum and colon. Additionally, in the ileum samples, *Bacteroides* in the sham-operated group were significantly increased compared with the normal group (*F* = 124.1, *P* < 0.0001), while *Escherichia-Shigella* was significantly decreased (*F* = 9.586, *P* < 0.01); the abundance of *Lachnospiraceae NK4A136 group* in the model group was significantly increased compared with the sham-operated group (*F* = 14.27, *P* < 0.001), while the abundance of *Bacteroides* was also significantly decreased (*F* = 124.1, *P* < 0.0001). In the colon sample, the *Lactobacillus* of the sham-operated group was significantly decreased compared with the normal group (*F* = 16.61, *P* < 0.05), and the abundance of *Lachnospiraceae NK4A136 group* in the model group was significantly increased compared with the sham-operated group (*F* = 14.27, *P* < 0.01).

**FIGURE 5:**
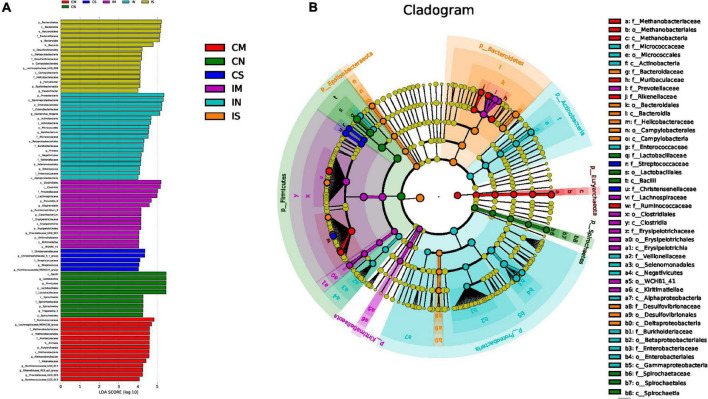
Effect of surgery and Aβ (1-42) injection on the composition of the significantly enriched species. **(A)** LDA score of LEfse (LDA > 4.0), used to show the significantly enriched species in each group and their degree of importance. **(B)** Cladogram of LEfse, used to show the evolutionary laws of species branching that play an important role in each group of samples. IN: the ileal microbiota of the normal group; IS: the ileal microbiota of the sham-operated group; IM: the ileal microbiota of the model group; CN: the colonic microbiota of the normal group; CS: the colonic microbiota of the sham-operated group; CM: the colonic microbiota of the model group.

LEfSe analysis enables comparison between multiple groups to find biomarkers with significant differences in abundance between groups. LEfSe analysis was used to further identify the species with significant differences in three groups (Figure 5) and to show similar findings. In the normal rats, Proteobacteria, Actinobacteria, *Escherichia-Shigella*, etc., are enriched in the ileum, and Firmicutes, *Lactobacillus*, etc., are enriched in the colon. In the sham-operated rats, Bacteroidetes and *Bacteroides* were enriched in the ileum, but there was no enrichment of high abundance microbiota in the colon. In the model rats, *Lachnospiraceae NK4A136 group* was enriched in the colon.

## Discussion

The Morris water maze is currently recognized as a classic experiment for studying spatial learning and memory in rodents. It is the most essential behavioral experiment for evaluating AD animal models (Pereira and Burwell, 2015). The behavioral results of this study showed significant changes in the water maze experiment in the model group rats compared with the normal and sham-operated groups, which indicates that bilateral ventricular injection of Aβ (1-42) impaired memory and cognitive functions in rats and successfully established the rat model of AD. Additionally, the significant increase in the content of Aβ (1-42) in the hippocampus of the model group also confirmed this.

The gut microbiota in the intestine is spatially heterogeneous, and different communities were observed along the length of the intestinal tract (Choi et al., 2014). There are also many previous reports on the segmental distribution of gut microbiota in mice, chickens, and pigs (Looft et al., 2014; Zhao et al., 2015). In this study, we found that the ileal and colonic microbiota were significantly different in terms of diversity and species by analyzing the α-diversity, β-diversity, and community composition of the ileal and colonic microbiota of normal rats. These results indicate that the ileal and colonic microbiota of normal SD rats also showed spatial heterogeneity, and the number of species and diversity of the ileal microbiota were significantly higher than that of the colonic microbiota.

In the process of exploring the difference between ileal and colonic microbiota of AD model rats, we found an interesting phenomenon: several indices of the ileal and colonic microbiota of normal rats were significantly different, showing spatial heterogeneity, while the ileal microbiota of rats in the sham-operated group, in which only surgery was conducted, showed reduced diversity and altered community structure, making the differences between the ileal and colonic microbiota narrow. And finally there were no significant differences between the ileal and colonic microbiota in Chao1 and Shannon index, the relative abundance of dominant phyla and genera of rats in the model group injected with Aβ (1-42). Therefore, we hypothesized that both the surgery and the injection of Aβ (1-42) caused the alteration of the gut microbiota.

Gut microbiota imbalance often occurs after major surgery or intestinal surgery and in critically ill patients (Earley et al., 2015; Mondot et al., 2015; Hamilton et al., 2020; Wang et al., 2020, 2021). Brain injury alters the composition of the intestinal microbiota, decreasing the intestinal microbial diversity (Wang et al., 2021). This phenomenon was reconfirmed using changes in the microbiota of sham-operated rats in the current study. Additionally, it was shown that traumatic frontal lobe knockout brain injury could lead to extensive changes in intestinal structures and function, affecting intestinal permeability (Feighery et al., 2008; Sundman et al., 2017). Particularly, the surgery in this study significantly increased the relative abundance of Bacteroidetes in sham-operated rats and significantly decreased the relative abundance of Proteobacteria and Actinobacteria, suggesting that it may be related to the effects of surgery on the gut microbiota. Additionally, the surgery also significantly increased the relative abundance of *Bacteroides* and significantly decreased the abundance of *Escherichia-Shigella*.

The departure of gut microbiota from a healthy state was considered a marker of disease onset and progression (Gilbert et al., 2016; Lynch and Pedersen, 2016). In recent years, many people have paid more attention to the study of gut microbiota and AD. Harach et al. (2017) found that the abundance of *Firmicutes*, *Verrucomicrobia, Proteobacteria*, and *Actinomycota* in the gut microbiota of AD model mice of the same age was significantly lower than the wild-type mice, while the abundance of *Bacteroidetes* was significantly higher. The gut microbiota plays an essential role in the regulation of neuroinflammation and neurodegeneration in AD (Goyal et al., 2021).

Senile plaques composed of β-amyloid protein (Aβ) are one of the classic pathological changes of AD. Harach et al. (2017) compared the Aβ content of sterilely-raised amyloid precursor protein transgenic AD model (APP/PS1) mice and conventionally raised APP/PS1 mice. They found that the Aβ content of germ-free mice was reduced compared with conventionally raised mice. Additionally, they also transplanted the microbiota of conventionally raised wild-type mice and APP/PS1 mice into sterilely-raised APP/PS1 mice, and discovered that the colonization of germ-free APP/PS1 mice with microbiota from conventionally raised APP/PS1 mice increased cerebral Aβ pathology, and the amount of Aβ deposition was similar to that of conventionally raised APP/PS1 mice, while colonization with microbiota from wild-type mice was less effective in increasing cerebral Aβ levels. Another study has shown that long-term treatment of APP/PS1 mice with broad-spectrum antibiotics will significantly reduce the diversity of gut microbiota and reduce Aβ plaque deposition in the cerebral cortex and hippocampus of mice (Minter et al., 2016). These findings confirmed that the gut microbiota can affect the production and deposition of Aβ.

In this study, we found that Aβ deposition in the hippocampus of the AD rat model established by bilateral intracerebroventricular injection of Aβ (1-42) was significantly increased. Concurrently, compared with the sham-operated mice, the colony structure of the ileum was significantly changed, the number and diversity of the colonic microbiota was also significantly increased. Therefore, we believe that artificially injecting Aβ into the brain of animals can also cause disorders of the gut microbiota. Particular, injection of Aβ (1-42) significantly increased *Lachnospiraceae NK4A136 group* in the ileum and colon of model rats. *Lachnospiraceae NK4A136 group* represents a kind of bacteria that produces butyrate, which has been found to maintain the integrity of the mouse intestinal barrier and is negatively correlated with intestinal permeability (Hu et al., 2019). As one of the main SCFAs produced by the microbiota, butyrate is essential in maintaining the health of the gastrointestinal tract because it can enhance the integrity of the epithelial barrier and inhibit inflammation (Hamer et al., 2008). Notably, the significant increase in the abundance of *Lachnospiraceae NK4A136 group* also highlights its underlying mechanism in AD lesions.

## Conclusion

In conclusion, this study used 16S rRNA high-throughput sequencing to compare and analyze the changes in the ileal and colonic microbiota of Alzheimer’s disease model rats established using bilateral intraventricular injection of Aβ (1-42), indicating that the microbiota of different intestinal segments of normal rats presents spatial heterogeneity. Both the surgery and injection of Aβ caused various degrees of disturbances in the ileal and colonic microbiota of rats. Particular, more in-depth analysis and discussion of the specific changes of the ileal and colonic microbiota during the modeling process was conducted, which can provide clues for a clearer understanding of the underlying mechanisms of AD lesions. This study allows us to clearly understand the impact of AD lesions on the different gut microbiota and provide more possibilities for intervention in AD from the perspective of gut microbiota. Disturbance of the gut microbiota may be of great significance to the pathogenesis of AD based on the brain-gut axis, and further studies are needed to confirm the connection between surgery, Aβ (1-42) injection, and disturbance of the gut microbiota. Also, whether the effect of Aβ (1-42) induced AD on gut microbiota between males and females is distinct needs to be further investigated.

## Data Availability Statement

The datasets generated for this study can be found in the NCBI SRA data with accession number PRJNA762025.

## Ethics Statement

The animal study was reviewed and approved by the Animal Ethics Committee of Southwest Jiaotong University (No. SWJTU-2010-001).

## Author Contributions

ZY and TZ conceived the project and designed the experiments. QX, LW, GW, XZ, YL, WX, TLZ, YF, CLC, CX, CC, YC, and QY performed the experiments. QX, LW, and GW wrote the manuscript. All authors read and approved the manuscript.

## Conflict of Interest

The authors declare that the research was conducted in the absence of any commercial or financial relationships that could be construed as a potential conflict of interest.

## Publisher’s Note

All claims expressed in this article are solely those of the authors and do not necessarily represent those of their affiliated organizations, or those of the publisher, the editors and the reviewers. Any product that may be evaluated in this article, or claim that may be made by its manufacturer, is not guaranteed or endorsed by the publisher.

## Abbreviations

AD: Alzheimer’s disease
Aβ: β-amyloid
BDNF: brain derived neurotrophic factor
5-HT: 5-hydroxytryptamine
SPF: specific pathogen free
MWM: morris water maze
OTU: operational taxonomic unit
ANOVA: one-way analysis of variance
LSD: least significant difference
HE: hematoxylin-eosin
CA: cornu ammonis
DG: dentate gyrus
PCoA: principal co-ordinates analysis
PCR: polymerase chain reaction
SCFAs: short chain fatty acids.
